# Dual Inhibition of BMP and WNT Signals Promotes Pancreatic Differentiation from Human Pluripotent Stem Cells

**DOI:** 10.1155/2019/5026793

**Published:** 2019-12-01

**Authors:** Mengtian Tan, Lai Jiang, Yinglei Li, Wei Jiang

**Affiliations:** ^1^Department of Biological Repositories, Zhongnan Hospital & Medical Research Institute, Wuhan University, Wuhan 430071, China; ^2^Affiliated Hospital of Hebei University of Engineering, Handan 056038, China; ^3^Hubei Provincial Key Laboratory of Developmentally Originated Disease, Wuhan 430071, China

## Abstract

Pathological or functional loss of pancreatic beta cells is the cause of diabetes. Understanding how signaling pathways regulate pancreatic lineage and searching for combinations of signal modulators to promote pancreatic differentiation will definitely facilitate the robust generation of functional beta cells for curing hyperglycemia. In this study, we first tested the effect of several potent BMP inhibitors on pancreatic differentiation using human embryonic stem cells. Next, we examined the endodermal lineage bias upon potent BMP inhibitor treatment and further checked the crosstalk between signal pathways governing endodermal lineage determination. Furthermore, we improved current pancreatic differentiation system based on the signaling pathway study. Finally, we used human-induced pluripotent stem cells to validate our finding. We found BMP inhibitors indeed not only blocked hepatic lineage but also impeded intestinal lineage from human definitive endoderm unexpectedly. Signaling pathway analysis indicated potent BMP inhibitor resulted in the decrease of WNT signal activity and inhibition of WNT could contribute to the improved pancreatic differentiation. Herein, we combined the dual inhibition of BMP and WNT signaling and greatly enhanced human pancreatic progenitor differentiation as well as beta cell generation from both embryonic stem cells and induced pluripotent stem cells. Conclusively, our present work identified the crosstalk between the BMP and WNT signal pathways during human endoderm patterning and pancreas specification, and provided an improved *in vitro* pancreatic directed differentiation protocol from human pluripotent stem cells.

## 1. Introduction

Diabetes mellitus is characterized by chronic hyperglycemia due to the loss of either beta cell mass or beta cell function and could lead to severe metabolic syndrome. There are around 425 million diabetes patients in the world, and the number is increasing according to the International Diabetes Federation (IDF) 2017 report. Traditional methods of treating diabetes include burdensome daily insulin-sensitizing drugs or insulin injection, which can only alleviate symptoms of hyperglycemia, but cannot maintain normaglycemia continually and thus fail to fundamentally cure diabetes. Islet transplantation provides an effective and reliable strategy to replace the damaged cells, but is largely limited by the shortage of cadaveric islet source [1].

Human pluripotent stem cells, including embryonic stem cells (ESCs) and induced pluripotent stem cells (iPSCs), could form multiple cell types and tissues composing of our body [2, 3]. Therefore, production of functional beta cells from human ESCs or iPSCs could be a promising choice for the cell replacement therapy of diabetes. A lot of efforts have been put in the last decades to direct human pluripotent stem cells to differentiate into functional beta cells *in vitro* and remarkable progresses have been recently achieved [4–9]. In order to generate robust beta cells from ESCs which represent the embryonic epiblast stage, pancreatic specification from differentiated definitive endoderm is a key point for the subsequent step [10]. The Nodal signaling pathway has been revealed as the main regulator of endoderm generation, but further endoderm patterning is more complicated and lacks of detailed studies in terms of signal combinations regulating individual endodermal lineage [11]. Retinoic acid (RA) is a well-known pathway to be utilized to direct pancreas specification, which also facilitates liver progenitor development [5, 12]. Bone morphogenetic protein (BMP) is required for hepatic specification from definitive endoderm both in human and mouse [13, 14]. NOGGIN, an inhibitory protein of BMP signaling, is thus utilized in pancreatic differentiation together with RA [4, 5, 8]. In addition, more potent compounds targeting the same pathway have been reported, such as retinoid analog TTNPB to replace the endogenous version *all-trans* RA [15] and LDN for NOGGIN [6, 7].

Previously, we have established a chemically defined protocol to direct human ESCs and iPSCs to differentiate into pancreatic lineage [5, 9, 16], and performed RNA sequencing analysis which pointed out BMP signaling as a downregulated pathway during pancreatic lineage specification from human ESC-derived definitive endoderm [17]. Therefore, here we tested several more stable and powerful chemical compounds targeting the BMP pathway to further promote pancreatic differentiation efficiency, and identified LDN193189 and K02288 indeed improving PDX1-positive pancreatic progenitor differentiation while surprisingly decreased CDX2-positive population. Since CDX2-positive cells represent intestine/colon lineages which are usually driven by the WNT signaling pathway, we had examined the crosstalk between the BMP and WNT signal pathways. Our data suggested that BMP inhibitors led to lower WNT activity and suppressed WNT signaling facilitated pancreatic lineage differentiation. Finally, we combined the dual treatment and observed the dual inhibition of BMP and WNT largely promoted pancreatic progenitor differentiation and beta cell generation.

## 2. Materials and Methods

### 2.1. Cell Culture and Differentiation

Human ESC line HUES8 [17] and iPSC line PGP1 [18] were cultured on Matrigel (BD Biosciences)-coated tissue culture plates with mTeSR1 medium accordingly (STEMCELL Technologies). For regular passaging, cells were split around 1 : 10 every 4-5 days with accutase for HUES8 or versene (Invitrogen) for PGP1, respectively.

For endodermal and pancreatic differentiation, we utilized previous protocols [5, 6, 16] with some modifications. First, human ESCs or iPSCs were seeded at the density of 30,000 to 50,000 cells per well for 24-well plate precoated with Matrigel. For induction of definitive endoderm, cells were treated with 100 ng/mL Activin A and 50 ng/mL Wnt3a recombinant protein for 1-2 days in 1 : 1 IMDM (Gibco) and F12 (Gibco) medium supplemented with 1% BSA, 1× B27 without vitamin A, and 1% penicillin-streptomycin (Invitrogen) and then followed by another 2 days with Wnt3a withdrawing. Then, differentiated definitive endoderm cells were cultured in MCDB131 (Sigma) for pancreatic progenitor differentiation, supplemented with 1.5 g/L sodium bicarbonate, 10 mM glucose (Invitrogen), 0.5% BSA, 0.25 mM ascorbic acid, 1× ITS-X, 1× GlutaMAX, and 1% penicillin-streptomycin. In addition, 50 ng/mL KGF, 0.5 *μ*M SANT1, 100 nM TTNPB, and 500 nM PDBU were added to this basal medium for 8 days, and with different individual BMP inhibitors (2 *μ*M K02288, 200 nM LDN193189, 2 *μ*M LDN214117, 2 *μ*M Dorsomorphin, 100 ng/mL Noggin, and individual WNT inhibitor (2 *μ*M XAV-939 or 2 *μ*M IWR-1). For following beta cell differentiation, differentiated pancreatic progenitor cells were cultured in MCDB131 with 20 mM glucose and 2% BSA, 1.5 g/L sodium bicarbonate, 0.05 mM ascorbic acid, 1× ITS-X, 1× GlutaMAX, and 1% penicillin-streptomycin. In addition, 200 nM LDN193189, 1 *μ*M T3, 10 *μ*M Repsox, 10 *μ*M YO-01027, and 10 *μ*M zinc sulfate were added for the first 5 days. In the last 5 days, cells were cultured in MCDB131 with 20 mM glucose and 2% BSA, 1.5 g/L sodium bicarbonate, 1× GlutaMAX, 1% penicillin-streptomycin, and 0.05 mM ascorbic acid. In addition, 1 *μ*M T3, 10 *μ*M Repsox, 10 *μ*M Trolox, 10 *μ*g/mL heparin sodium, 2 *μ*M R428, 10 *μ*M zinc sulfate, and 10 mM N-cys were added. All the chemical compounds are commercially available and the detailed information is listed in [Supplementary-material supplementary-material-1].

### 2.2. Western Blotting Analysis

Cells were harvested and lysed on ice with RIPA buffer supplemented with cOmplete Tablets EDTA-free EASYpack (Roche). Proteins (20 mg) were separated by 10% sodium dodecyl sulfate-poly-acrylamide gel electrophoresis and transferred onto a nitrocellulose membrane (Millipore). The membrane was blocked with 5% skim milk in TBS buffer with 0.1% Tween-20 and incubated with antibodies against beta-catenin, active beta-catenin, goat anti-human PDX1, or GAPDH overnight at 4°C. Then, the membrane was washed and incubated with secondary antibodies at room temperature for 2 hours. After washing, the membrane was visualized with SuperSignal® West Pico Chemiluminescent Substrate (Thermo Fisher).

### 2.3. Immunofluorescence Staining

Cells were washed with PBS and fixed in 4% PFA for 20 minutes at room temperature. After washed with PBS for three times, the cells were blocked and permeabilized with PBS containing 10% normal donkey serum and 0.1% Triton X-100 for 2 hours at room temperature. Then, diluted primary antibodies were added and incubated overnight at 4°C. The primary antibodies used included goat anti-human PDX1, rabbit anti-human PDX1, rabbit anti-human AFP, rabbit anti-human CDX2, mouse anti-human CHGA (Chromogranin A), rabbit anti-human CHGA, rabbit anti-human GLUCAGON, mouse anti-human INSULIN, rabbit anti-human INSULIN, rat anti-C-Peptide, mouse anti-NKX6-1, mouse anti-pY489-beta-catenine, rabbit anti-beta-catenin, mouse anti-Pro-INSULIN, and goat anti-human SOX2. After washing with PBS for five times, the cells were incubated with diluted TRITC-or 488-conjugated donkey anti-rabbit, goat, and mouse IgG secondary antibodies at room temperature in the dark for 2 hours. The nuclei were counterstained with 2‰ 4,6-diamidino-2-phenylindole (DAPI, Sigma, Cat #10236276001). The detailed information of all the used antibodies is listed in [Supplementary-material supplementary-material-1].

### 2.4. Flow Cytometry

Cells were dissociated with 0.05% trypsin-EDTA solution and incubated at 37°C for 5-10 minutes. Cells were suspended in 1 mL FACS Wash Buffer and collected by centrifuging at 1000 rpm for 5 minutes. Then, the cells were suspended in commercially available fixation/permeabilization solution (R&D) for 45 minutes at 4°C and then washed with Perm/Wash solution. Primary antibodies PDX1, CDX2, and AFP or isotype control IgG were incubated with suspended single cells in 200 *μ*L Perm/Wash overnight at 4°C. After wash, secondary antibodies were incubated at room temperature for 2 hours in dark. Then, samples were transferred in a total volume of 300-500 *μ*L FACS Buffer and analyzed with the flow cytometer (FACSCelesta).

### 2.5. RNA Isolation and Quantitative RT-PCR

RNA was extracted using Hipure Total RNA Mini kit (Magen), and cDNA was generated immediately following RNA isolation using All-in-One cDNA Synthesis SuperMix (Bimake) according to the manufacturer's manual. The sequences of quantitative PCR primers are listed as below: *PDX1* (TTAGGATGTGGACGTAATTCCTGTT, GGCCACTGTGCTTGTCTTCA); *INSULIN* (GCAGCCTTTGTGAACCAACAC, CCCCGCACACTAGGTAGAGA); *NKX6-1* (AGACCCACTTTTTCCGGACA, CCAACGAATAGGCCAAACGA); *NGN3* (CTAAGAGCGAGTTGGCACTGA, GAGGTTGTGCATTCGATTGCG); *NEROUD* (ACCAAATCGTACAGCGAGAGT, CTCGTCCTGAGAACTGAGACA); *AXIN2* (CTGGTGCAAAGACATAGCCA, AGTGTGAGGTCCACGGAAAC); *WNT3* (AGTTGCTTGGGGACCAGG, CTCGCTGGCTACCCAATTT); *CDX2* (CTGGAGCTGGAGAAGGAGTTTC, ATTTTAACCTGCCTCTCAGAGAGC); *AFP* (CCCGAACTTTCCAAGCCATA, GTACATGGGCCACATCCAGG); *MYC* (GACTGTATGTGGAGCGGTTTC, CGTTGAGCGGGTAGGGA); and *TWIST1* (CAGCGGGTCATGGCTAACG, CAGGACCTGGTACAGGAAGTCGA). Quantitative PCR was performed on CFX384 module (Bio-Rad) using 2X SYBR Green qPCR kit (Bimake).

### 2.6. Glucose-Stimulated INSULIN/C-Peptide Secretion

Differentiated beta cells at the final stage were washed with Krebs buffer containing 2 mM glucose for more than 2 hours to remove the residual INSULIN. After washing three times with Krebs buffer, cells were incubated with low glucose (2 mM) in Krebs buffer for 30 minutes, and supernatant was collected; then, cells were further washed three times in Krebs buffer and incubated with high glucose (20 mM) or 30 mM KCl in Krebs buffer for another 30 minutes, and then, supernatant was collected. All the supernatant samples were processed to analyze the concentration of INSULIN by Ultrasensitive Insulin ELISA (Mercodia) and C-Peptide by Ultrasensitive C-Peptide ELISA (Mercodia). For normalization, protein content of the cell lysate was determined using the BCA kit (Thermo Fisher).

### 2.7. Statistical Analysis

All experiments were performed at least three times. The data are presented as the mean ± SD. All statistical analyses were performed using SPSS 22.0 software. Student's *t*-test was conducted between two groups. Statistical significance was set at *p* values < 0.05.

## 3. Results

### 3.1. Compound Test for BMP Inhibitors to Improve the Generation of PDX1-Positive Cells

Our previous study profiled the transcriptome dynamics of entire pancreatic lineage including undifferentiated ESCs, purified definitive endoderm, and pancreatic progenitors, as well as alpha and beta cells from primary human islets [17]. By focusing on the pancreatic lineage specification stage (i.e., from definitive endoderm to pancreatic progenitor), we found the BMP signaling pathway significantly enriched [17]. NOGGIN, as a well-recognized BMP antagonist, has been broadly used in previous protocols [4, 5, 8] and effectively directed pancreatic differentiation. However, considering the cost and efficacy, we performed a small test for chemical compounds inhibiting the BMP and TGFbeta signaling pathway, including LDN193189 (Selleck, S2618) targeting ALK2/3 which was already used in a previous report [6], K02288 (Selleck, S7359) blocking ALK1/2/3/6, LDN214117 (Selleck, S7627) exclusively targeting ALK2, and Dorsomorphin (Compound C, Selleck, S7840) recognized as BMP inhibitor and AMPK inhibitor. NOGGIN (Peprotech, 120-10C) and no BMP inhibitor were served as control.

**Figure 1:**
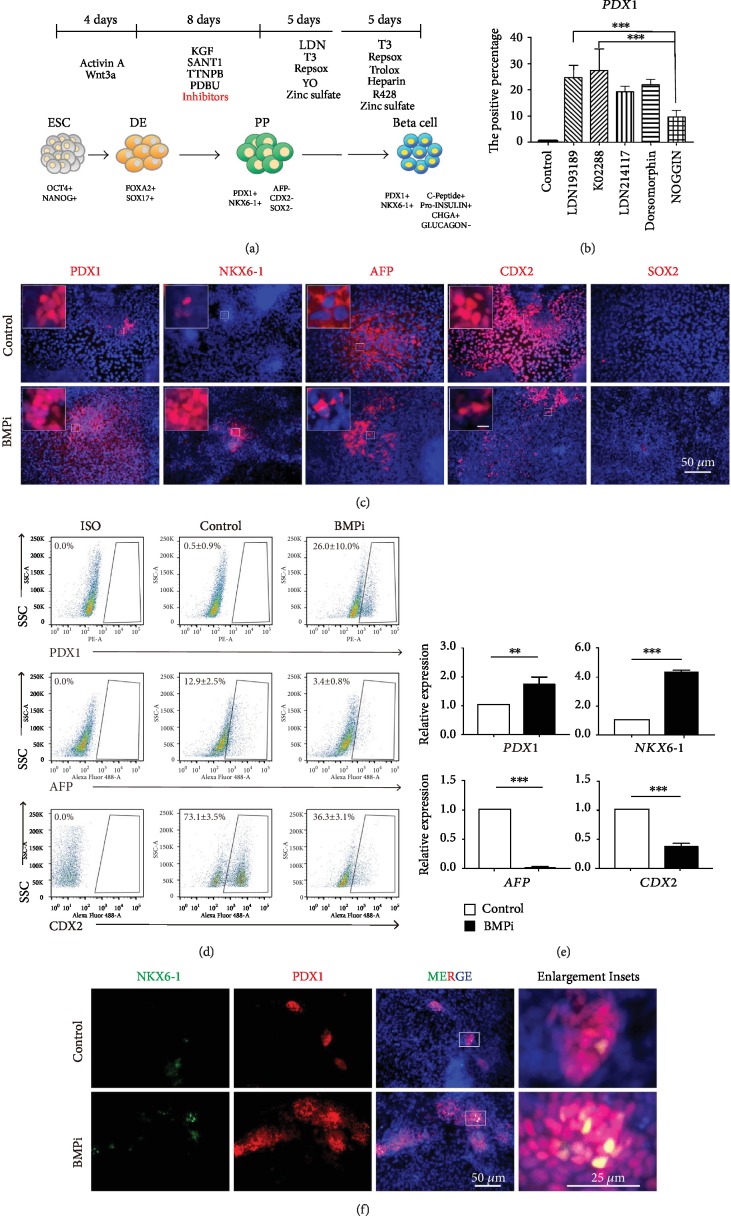
The endodermal lineage differentiation while BMP inhibited. (a) The schematic diagram displayed differentiation protocol (ESC: embryonic stem cells; DE: definitive endoderm stage; PP: pancreatic progenitors; beta cell: pancreatic beta cell stage). (b) The statistics of PDX1-positive cells determined by intracellular flow cytometry with different BMP inhibitors. The control group had no inhibitor of BMP. LDN193189 (targeting ALK2/3), K02288 (targeting ALK1/2/3/6), LDN214117 (targeting ALK2), Dorsmorphin (targeting BMP and AMPK), and NOGGIN were added in each experimental group at PP stage (*n* = 3). (c) Immunofluorescence results exhibited the control group (no BMP inhibitor) and BMPi (shown for LDN193189) in PP stage with pancreatic markers PDX1 and NKX6-1, and liver marker AFP, intestine marker CDX2, and gastric marker SOX2. DAPI (blue) represented all cells for internal control. Scale bars for enlargement inset, 50 *μ*m. SOX2 antibody had been validated previously. (d) Flow cytometry showed the analysis of different endodermal lineages (PDX1 for pancreatic lineage; AFP for hepatic lineage; CDX2 for intestinal lineage) (*n* = 3). (e) mRNA expression levels of *PDX1*, *NKX6-1*, *AFP*, and *CDX2* in the control group and BMPi group. ∗∗ represented for *p* value < 0.01 and ∗∗∗ for *p* value < 0.001 by Student's *t* test (*n* = 3). (f) Costaining PDX1 and NKX6-1 represented bona fide pancreatic progenitors in BMPi condition.

We used human ESC line HUES8 with an integrated protocol based on our previous study [16, 17] and recent report [6]. After generating definitive endoderm, we treated with different compound inhibitors in the same condition (Figure 1(a)), and evaluated the pancreatic differentiation effect. PDX1 is one of the most important transcription factors to specify pancreatic fate and beta cell maturation [19]; herein, we examined the ratio of PDX1-positive cells at progenitor stage by immunofluorescence assay and later quantitatively confirmed by flow cytometric analysis. The immunostaining data indicated LDN193189 treatment induced the most PDX1-positive cells ([Supplementary-material supplementary-material-1]). The flow cytometric analysis revealed that K02288 and LDN193189 were vigorous candidates with around 25% PDX1-positive cells, compared to less than 10% for NOGGIN (Figure 1(b), [Supplementary-material supplementary-material-1]). Consistently, we observed that hepatic marker AFP exhibited a significant decrease upon treated with various BMP inhibitors ([Supplementary-material supplementary-material-1]).

### 3.2. The Endodermal Lineage Patterning while Inhibiting BMP Signal

We further verified the effect of BMP inhibitor (BMPi for short) LDN193189 (Figure 1) and K02288 ([Supplementary-material supplementary-material-1]) on endodermal lineage specification from differentiated definitive endoderm. We observed that the percentage of PDX1-positive cells greatly increased after BMPi treatment by immunofluorescence (Figure 1(c), [Supplementary-material supplementary-material-1]), which was further confirmed by flow cytometry analysis (Figure 1(d), [Supplementary-material supplementary-material-1]). Since NKX6-1-expressing cells within PDX1-positive population indicated the bona fide pancreatic progenitors [4, 6, 7], we also checked NKX6-1, showing upregulated expression in the BMPi treatment group both in protein and mRNA levels (Figures 1(c), 1(e), and 1(f), [Supplementary-material supplementary-material-1]).

Then, we investigated other nonpancreatic lineages in the differentiated endoderm derivatives after BMPi treatment. The pancreas is derived from embryonic gut tube, which also forms the lung, gastric fundus, liver, intestine, colon, and others. Since the liver, intestine, and stomach are derived from posterior foregut, the same as the pancreas [20], we determined the expression of hepatic marker AFP and intestinal marker CDX2 as well as gastric marker SOX2. The data indicated both the percentage of AFP-positive cells (Figures 1(c) and 1(d), [Supplementary-material supplementary-material-1] and [Supplementary-material supplementary-material-1]) and mRNA level of AFP (Figure 1(e), [Supplementary-material supplementary-material-1]) dramatically decreased as expected, as BMP is the major inducer of hepatic lineage [13, 14]. Surprisingly, the percentage of CDX2-positive cells also exhibited significant decrease from more than 70% to lower than 40% (Figure 1(d)), which was further validated by immunofluorescence analysis (Figure 1(c), [Supplementary-material supplementary-material-1]), as well as the RNA expression level (Figure 1(e), [Supplementary-material supplementary-material-1]). There was no significant change of SOX2-positive cells possibly due to the generally lower expression level (Figure 1(c)). The PDX1 and NKX6-1 coexpressing cells appeared upon BMP inhibition (Figure 1(f), [Supplementary-material supplementary-material-1]). Taken together, we observed increased pancreatic differentiation while decreased expected hepatic differentiation and unexpected intestinal differentiation after BMPi treatment.

### 3.3. BMP Inhibition Led to Suppressed WNT/Beta-Catenin Signaling Activity during Pancreatic Differentiation

**Figure 2:**
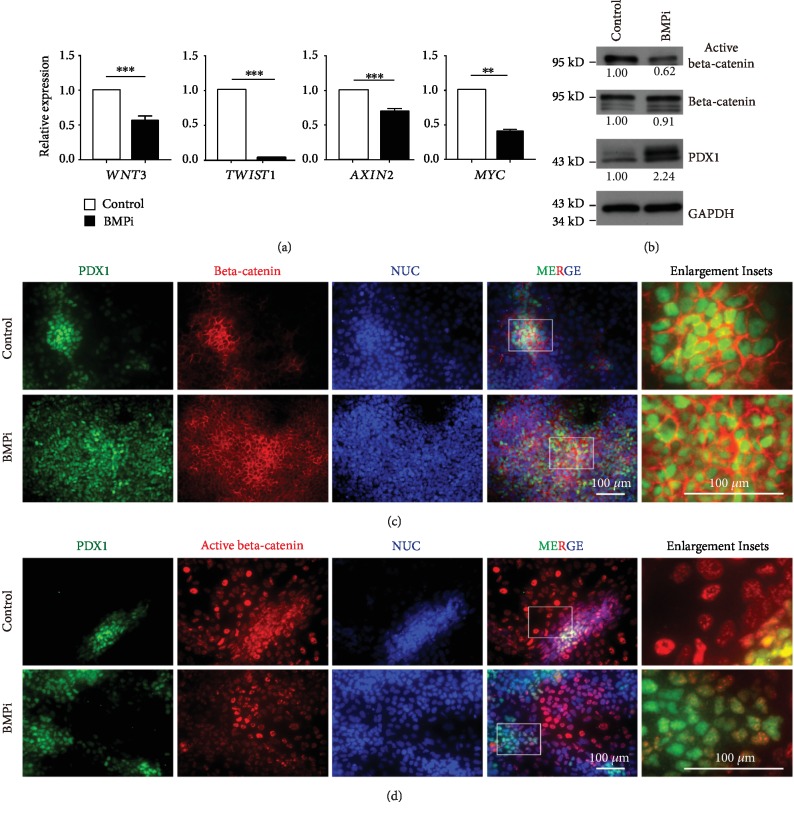
BMPi regulated pancreatic differentiation via decreased WNT activity. (a) The expression levels of WNT downstream genes (including *WNT3*, *TWIST1*, *AXIN2*, *MYC*) were checked between the control group and BMPi group (LDN193189) in PP stage. ∗∗ represented for *p* value < 0.01 and ∗∗∗ for *p* value < 0.001 by Student's *t*-test (*n* = 3). (b) Protein levels of PDX1, active beta-catenin, and total beta-catenin were analyzed by Western blotting in the control group and the BMP inhibitor group in PP (*n* = 3). (c, d) The protein of total beta-catenin and active beta-catenin were costained with PDX1 to show the intracellular localization.

To understand the result that BMPi promoted pancreatic differentiation and inhibited intestinal differentiation (Figure 1), we investigated whether inhibition of BMP signaling during pancreatic differentiation affected the WNT signaling pathway, which was considered as the major inducer of intestinal lineage [21]. First, we checked the expression levels of downstream genes to evaluate the activity of the WNT signaling pathway. The data showed all checked canonical WNT downstream genes, including *WNT3*, *TWIST1*, *AXIN2*, and *MYC*, and exhibited significant downregulation after inhibiting BMP (Figure 2(a), [Supplementary-material supplementary-material-1] for another BMP inhibitor K02288), indicating lower WNT signaling activity. Then, we checked the protein level and phosphorylation level of beta-catenin, the main effector of the WNT signaling pathway. Cytosolic beta-catenin phosphorylation/degradation is the essence of WNT signaling [22]. Phosphorylation of beta-catenin at serine 37 and serine 33 by GSK3 will lead to its ubiquitination and degradation [23], while hyperphosphorylation on tyrosine residues will make beta-catenin dissociate from N-cadherin and being on-state situation [24]. Thus, nonphosphorylated beta-catenin (Ser33/37/Thr41) or Y489-phosphorylated beta-catenin represents nuclear-bound activated form to determine WNT/beta-catenin activity. Therefore, we collected the cell lysate to examine protein expression and the results showed the BMPi treatment group exhibiting higher PDX1 protein level and lower active beta-catenin protein level albeit unchanged total beta-catenin level (Figure 2(b)). We further looked into the coexpression and subcellular localization of beta-catenin and active beta-catenin with PDX1, respectively (Figures 2(c) and 2(d), [Supplementary-material supplementary-material-1]). The total beta-catenin protein showed no difference in the control or BMPi group in both PDX1-positive cells and PDX1-negative cells (Figure 2(c)), as beta-catenin is not only the downstream of WNT signal but also the cytoskeletal structure protein with Ca2^+^-dependent CAMs [24]. In contrast, nucleus-located activated beta-catenin signals indeed decreased in BMPi condition compared with control (Figure 2(d), [Supplementary-material supplementary-material-1]). Furthermore, the PDX1-positive cells exhibited apparently much weaker staining of beta-catenin in the nucleus, suggesting lower activity of WNT signaling, while PDX1-negative cells had upregulated nucleus-located beta-catenin protein, suggesting higher activity of WNT signaling (Figure 2(d), [Supplementary-material supplementary-material-1]). Taken together, these data suggested the inhibition of BMP led to lower activity of the WNT signal pathway.

### 3.4. Direct Inhibition of WNT Could Promote Pancreatic Differentiation and Beta Cell Generation

**Figure 3:**
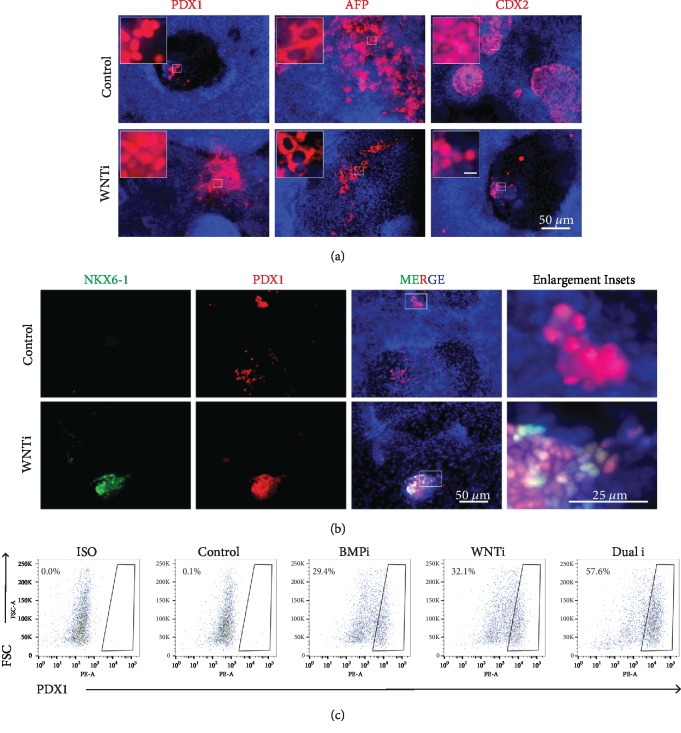
Inhibition of WNT could facilitate the generation of pancreatic progenitor cells. (a) Immunofluorescence results exhibited the control group (no BMP inhibitor) and WNTi (using WNT inhibitor XAV939) at PP stage with pancreatic marker PDX1, liver marker AFP, and intestine marker CDX2. DAPI (blue) represented all cells for internal control. Scale bars for enlargement inset, 50 *μ*m. (b) Costaining PDX1 and NKX6-1 represented bona fide pancreatic progenitors in WNTi (using WNT inhibitor IWR-1) condition. (c) Flow cytometry showed the analysis of PDX1-postive cells.

Since BMP inhibition improved pancreatic differentiation along with lower WNT/beta-catenin activity, we attempted to block WNT (labeled as WNTi) in pancreatic differentiation from definitive endoderm stage (Figure 1(a)). WNT inhibition alone by XAV939 or IWR-1 indeed showed improved effect in terms of promoting pancreatic lineage marker expression and decreasing liver and intestinal lineage marker expression (Figures 3(a) and 3(b)); however, dual inhibition exhibited much higher expression levels of pancreatic markers and more PDX1-positive cells (Figure 3(c)).

### 3.5. Dual Inhibition of BMP and WNT in Pancreatic Differentiation

**Figure 4:**
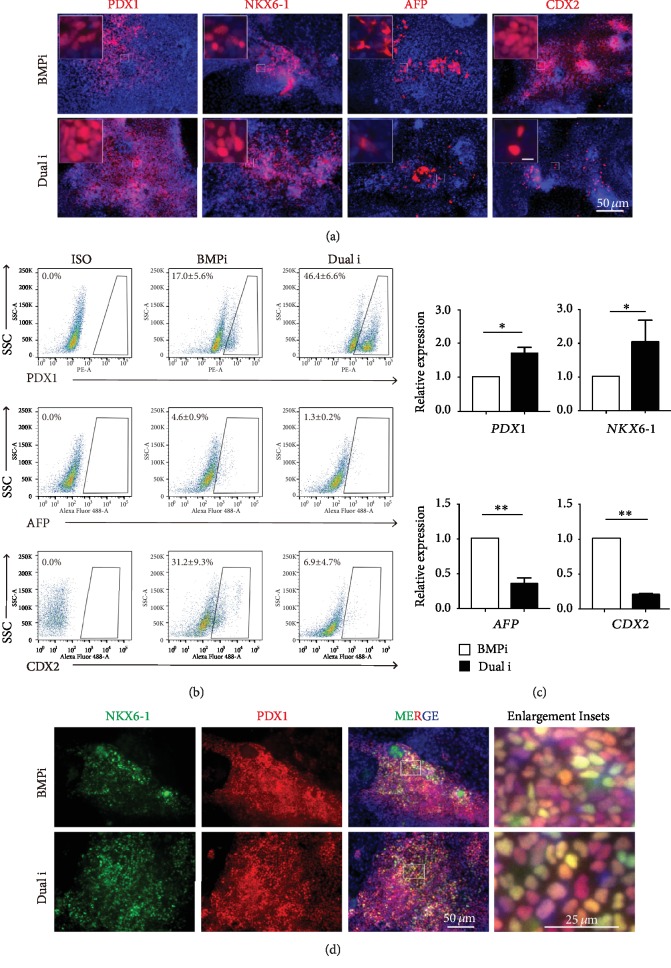
Further inhibition of WNT and BMP could promote pancreatic progenitor differentiation. (a) The immunofluorescence showed the BMPi (BMP inhibitor only) and “Dual i” (BMP inhibitor LDN193189 plus WNT inhibitor XAV939 together) groups with endodermal lineage marker in pancreatic progenitor stage. The expression levels of PDX1 and NKX6-1 (pancreatic lineage) were much higher in “Dual i,” while AFP (liver lineage) and CDX2 (intestine lineage) fell down in “Dual i.” Scale bars for enlargement inset, 50 *μ*m. (b) Flow cytometry showed the analysis of different endodermal lineage markers (PDX1, AFP, CDX2) between the BMPi and “Dual i” (*n = 3*). (c) mRNA expression of endodermal lineages (PDX1, NKX6-1, AFP, CDX2) analyzed in BMPi and “Dual i.” ∗ represented for *p* value < 0.05 and ∗∗ for *p* value < 0.01 by Student's *t*-test (*n* = 3). (d) Costaining PDX1 and NKX6-1 represented bona fide pancreatic progenitors in “Dual i” and BMPi conditions.

Since dual inhibition of BMP and WNT showed the best induction effect of pancreatic differentiation (Figure 3(c)), we looked into the combination of dual inhibition of BMP and WNT signals. Very dramatically, double inhibition of BMP and WNT signals (labeled as “Dual i”) largely increased the percentage of PDX1-positive cells up to about 50% (Figures 4(a)–4(c)). Meanwhile, we checked other endodermal lineages and found the mildly decreased AFP-positive hepatic lineage and dramatically lower CDX2-positive intestinal lineage (less than 10%) (Figures 4(a) and 4(b)). We also performed mRNA analysis and found “Dual i” indeed increased the *PDX1* and *NKX6-1* expression level and reduced *AFP* and *CDX2* levels (Figure 4(c)). More importantly, PDX1- and NKX6-1-double positive cells also significantly increased in “Dual i” (Figure 4(d)), indicating that bona fide pancreatic progenitors were capable of generating beta cells [4, 6, 7]. Our data together showed that the pancreatic lineage differentiation was greatly enhanced by the “Dual i” treatment at the expense of hepatic and intestinal lineage.

**Figure 5:**
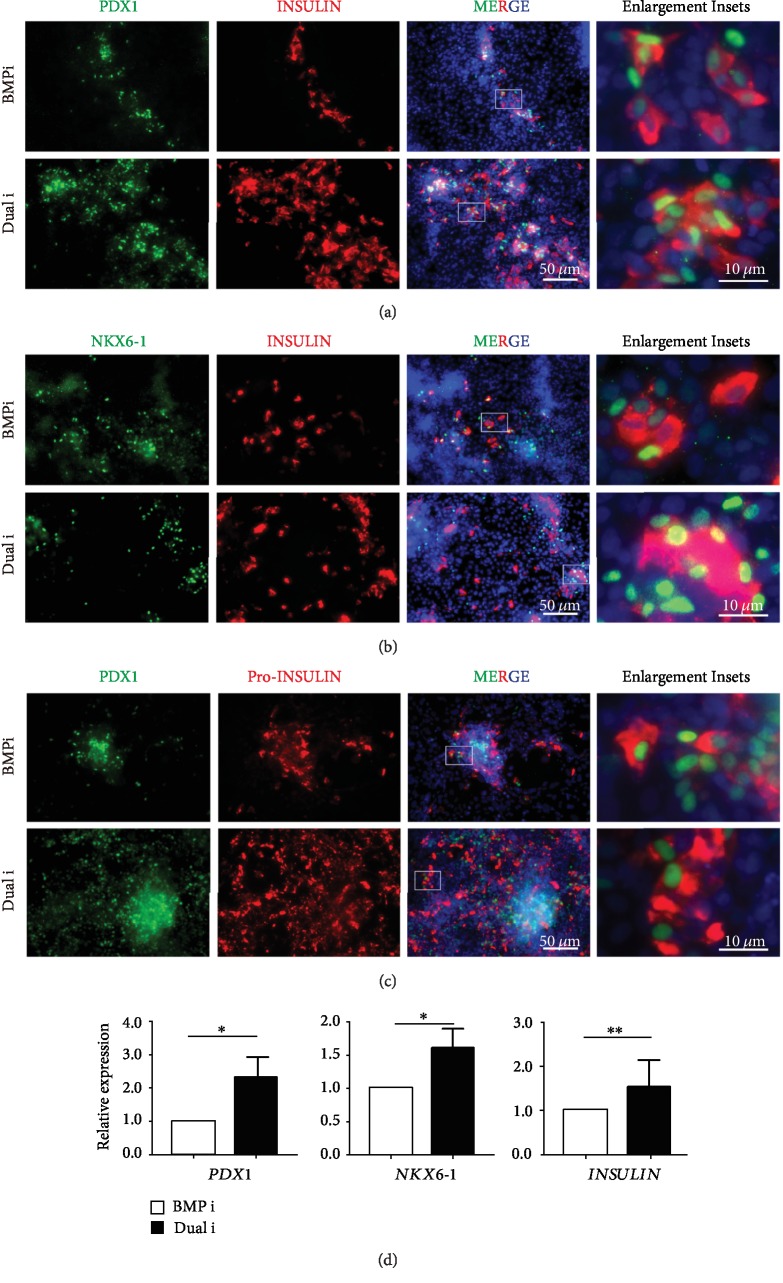
Dual inhibition of BMP and WNT facilitated beta cell differentiation. (a, b) Generation of high INSULIN (red color) and nucleus transcription factor PDX1, NKX6-1 (green color) copositive population showed in “Dual i” and BMPi. (c) PDX1 (green color) and *de novo* INSULIN production markers Pro-INSULIN (red color) were evaluated by immunofluorescence in beta cell stage. (d) mRNA expression of *PDX1*, *NKX6-1*, and *INSULIN* between BMPi and “Dual i” exhibited in beta cell stage. ∗ represented for *p* value < 0.05 and ∗∗ for *p* value < 0.01 by Student's *t*-test (*n* = 3).

We further checked whether the dual treatment of BMPi and WNTi could also give rise to better beta cell efficiency in the following beta cell differentiation condition. To evaluate the beta cell generation, we costained the functional marker INSULIN with transcription factors PDX1 and NKX6-1. Immunofluorescence data showed that dual inhibition resulted in the higher ratio of INSULIN-positive cells costained with PDX1 or NKX6-1 (Figures 5(a) and 5(b)). Since Pro-INSULIN is the precursor for INSULIN maturation and thus represents the *de novo* production of INSULIN, we examined the expression of Pro-INSULIN. The results showed the “Dual i” group resulting in much higher positive cells for Pro-INSULIN with PDX1 (Figure 5(c)). We further surveyed the RNA expression level and indeed dual inhibition treatment exhibited higher expression levels of *PDX1*, *NKX6-1*, and *INSULIN* genes (Figure 5(d)).

### 3.6. Dual Inhibition of BMP and WNT in Pancreatic Differentiation from Human iPSCs

**Figure 6:**
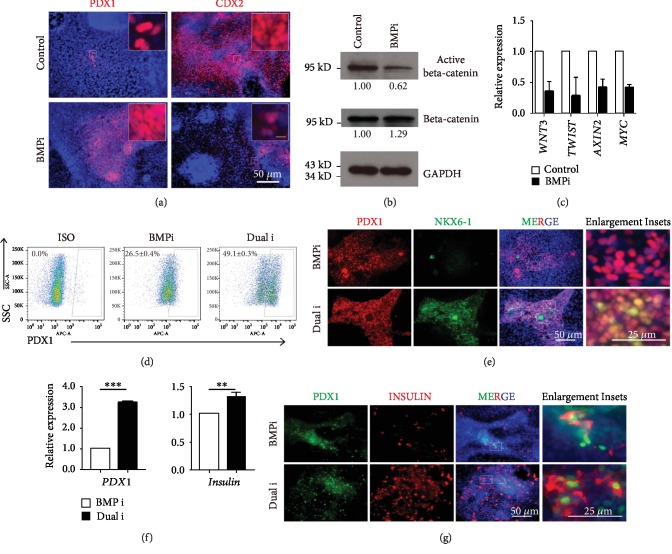
Dual inhibition of BMP and WNT promoted pancreatic differentiation of human iPSCs. (a) Immunofluorescence showed the PDX1 and CDX2 expressions upon treated with BMP inhibitor (LDN193189) compared to the control group. Scale bars for enlargement inset, 50 *μ*m. (b) Protein levels of active beta-catenin and total beta-catenin were analyzed with control and BMPi by Western blotting (*n* = 3). GAPDH served as a loading control. (c) qPCR analysis showed the expression levels of WNT downstream genes including *WNT3*, *TWIST1*, *AXIN2*, and *MYC* between control and BMPi (*n* = 3). (d) Flow cytometric analysis displayed PDX1 between the BMPi and “Dual i” in PGP1 pancreatic differentiation (*n* = 3). (e) Immunofluorescence showed the progenitor markers PDX1 and NKX6-1 coexpression between the BMPi and “Dual i” groups at PP stage. (f) mRNA expression level of *PDX1* and *INSULIN* between the BMPi and “Dual i” groups was revealed in beta cell stage. ∗∗ represented for *p* value < 0.01 and ∗∗∗ for *p* value < 0.001 by Student's *t*-test (*n* = 3). (g) Immunofluorescence showed the beta cell markers PDX1 and INSULIN coexpression at beta cell stage between the BMPi and “Dual i” groups.

We chose another cell line human iPSC PGP1 to evaluate the common role of the signaling crosstalk between BMP and WNT during pancreatic specification. We first generated definitive endoderm cells and treated with BMP inhibitor LDN193189 along with pancreatic specification condition. Similarly, LDN193189 significantly increased PDX1-positive cells and greatly decreased CDX2-positive cells as expected (Figure 6(a)). Consistent with this observation, the protein level of active beta-catenin in the BMPi group was significantly lower than the control group while there was no change for the total beta-catenin level (Figure 6(b)). The expression levels of canonical WNT target genes including *WNT3*, *TWIST1*, *AXIN2*, and *MYC* were downregulated in the BMPi group (Figure 6(c)). These data indicated lower WNT activity appearing upon BMP inhibition, which was the same as that we observed in human ESCs (Figure 2).

We further utilized the combination of dual inhibition of BMP and WNT signaling for pancreatic differentiation and performed the flow cytometric analysis. The result showed “Dual i” indeed increased the PDX1-positive cells in progenitor stage (Figure 6(d)). Consistently, immunostaining result indicated dual inhibition of BMP and WNT generating much higher PDX1-and NKX6-1-double positive cells at progenitor stage (Figure 6(e)). We further evaluated the beta cell differentiation efficiency by RT-qPCR and immunostaining assays. Dual inhibition largely improved the mRNA expression levels of *PDX1* and *INSULIN* (Figure 6(f)). Similarly, dual inhibition of BMP and WNT generated much higher PDX1- and INSULIN-double positive and PDX1-positive cells in iPSC (Figure 6(g)).

### 3.7. Functional Analysis of Differentiated Beta Cells by Dual Inhibition

**Figure 7:**
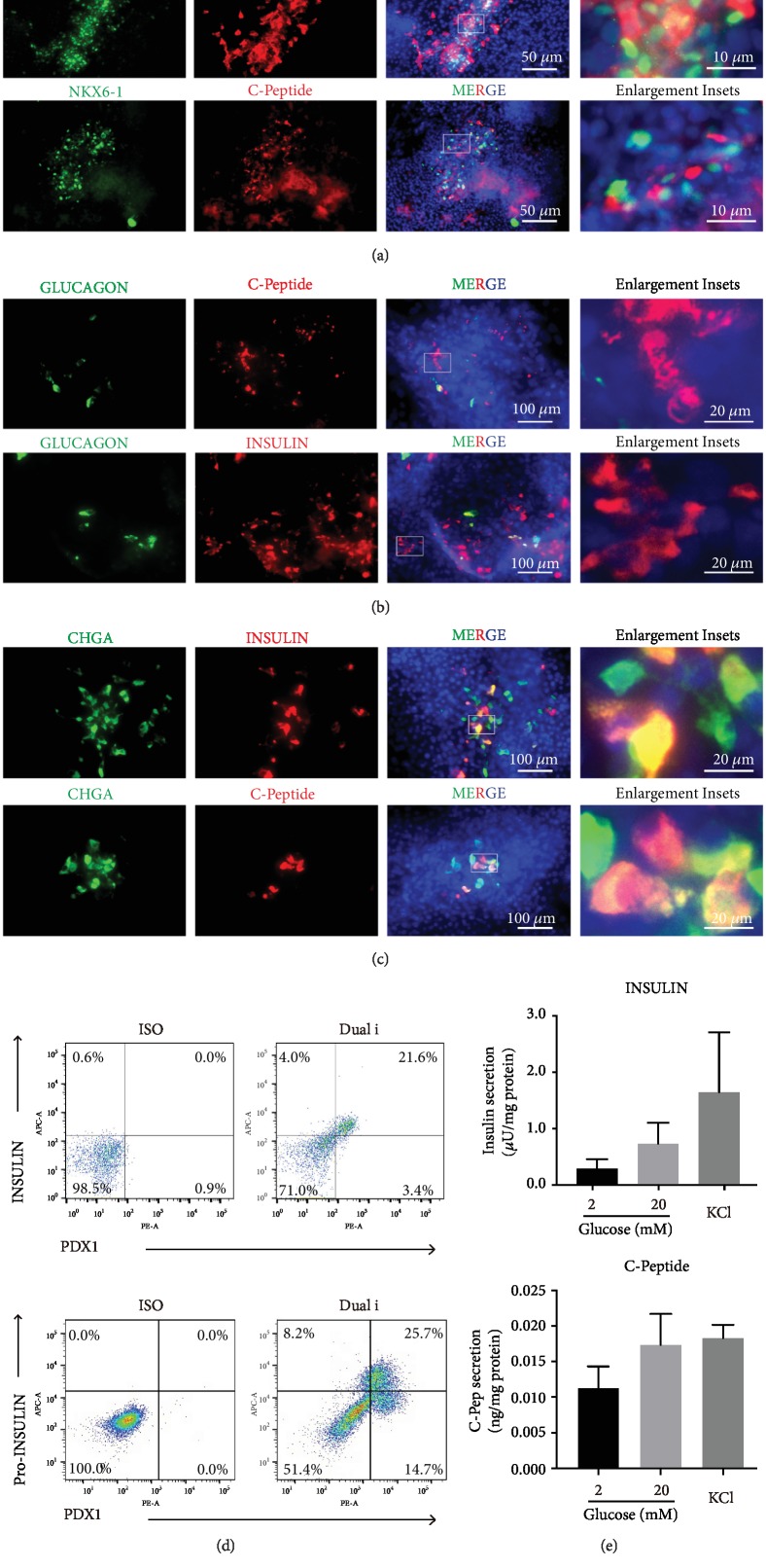
Functional analysis of differentiated beta cells by dual inhibition (a–c). Costaining of C-Peptide (red color) with nucleus transcription factor PDX1, NKX6-1 (green color) (a). Glucagon (green color) with INSULIN/C-Peptide (red color) (b). Chromogranin (CHGA, green color) with INSULIN/C-Peptide (red color) (c). Flow cytometry showed the analysis of PDX1 with INSULIN or C-Peptide at the final beta cell differentiation stage (d). The levels of secreted human INSULIN and C-Peptide of differentiated beta cells measured by ELISA upon challenging with 2 mM glucose, 20 mM glucose, and 30 mM KCl (e).

Since dual inhibition of BMP and WNT resulted in higher PDX1- or NKX6-1- with INSULIN- or Pro-INSULIN-double positive cells, we next examined the beta cell identity. First, we performed more costaining to confirm PDX1 and NKX6-1 coexpressed with C-Peptide (Figure 7(a)). In addition, the expression of INSULIN/C-Peptide was exclusive, because INSULIN/C-Peptide-positive cells were not expressing GLUCAGON (Figure 7(b)), but indeed costained with secretory protein CHGA (Chromogranin A) (Figure 7(c)). By further quantitative flow cytometric analysis, approximately 21% of cells coexpressed INSULIN and PDX1 in differentiated beta cells, and 25% of cells were PDX1 and Pro-INSULIN-double positive cells (Figure 7(d)). The most important functional feature of beta cells is the capacity to secret INSULIN response to stimuli such as glucose and KCl. We challenged the differentiated beta cells with 20 mM glucose and we found the differentiated cells secreted INSULIN upon high glucose stimulation with around 2.5 ± 1.3-fold than basal glucose treatment. C-Peptide analysis also indicated the differentiated cells released more C-Peptide in high glucose (1.5 ± 0.8-fold) and KCl (1.6 ± 1.9-fold) conditions compared to basal glucose level (Figure 7(e)). Taken together, the differentiated beta cells through dual inhibition of BMP and WNT indeed exhibited the characteristic of functional beta cells.

## 4. Discussion

Our present study reported an unexpected but important result that BMP inhibition could repress WNT activity during pancreatic differentiation (Figures 1 and 2, [Supplementary-material supplementary-material-1] and [Supplementary-material supplementary-material-1]) and dual inhibition treatment of BMP and WNT signals could greatly promote pancreatic differentiation from human ESCs (Figures 4 and 5) and iPSCs (Figure 6) as well. This finding not only facilitated the pancreatic beta cell generation for potential cell therapy and disease modeling but also highlighted the importance of crosstalk among signaling pathways in cell fate determination.

Endodermal organ patterning was a very complicated process. Primitive gut tube could be simply divided into foregut, midgut, and hindgut according to the anterior-posterior position. Later, foregut experienced a complicated fate determination process and formed the whole respiratory organ systems and the upper digestive system. In particular, the generation of the stomach, pancreas, liver, and intestine derived from a common pool of progenitors in the foregut endoderm, requiring the precise combinations of different signal pathways. BMP was not only required for hepatic specification from definitive endoderm both in human and mouse [13, 14] but also contributed to the posterization of gut tube patterning by upregulating the hindgut marker CDX2 and repressing the anterior gut marker SOX2 [25]. RA had been widely used to induce posterior foregut and pancreatic lineage [4–7, 9]. It had been reported RA treatment resulted in the expression of hepatic marker AFP when added directly to the Activin A-induced human ESCs [5, 12]. Therefore, the combination of RA and BMP inhibitor was expected to promote pancreatic lineage specification, which had been evidenced by multiple groups [4, 5, 8]. Our present data also demonstrated inhibition of BMP by various different chemicals greatly repressed hepatic lineage and facilitated pancreatic lineage differentiation in general (Figure 1, [Supplementary-material supplementary-material-1]).

Furthermore, our results revealed that BMP inhibition reduced WNT activity during human endodermal patterning (Figure 2, [Supplementary-material supplementary-material-1]). Consistent with our observation, there were several studies indicating the crosstalk between the BMP and WNT signal pathways in different animal models or developmental contexts. During caudal vein formation in zebrafish, BMP induced the expression of AGGF1, a beta-catenin transcriptional cofactor which activated beta-catenin and WNT activity [26]. Roarty and Serra demonstrated that TGFbeta regulated the expression of wingless-related protein family Wnt5a in tumor progression and metastasis [27]. Placencio and colleagues showed TGFbeta signaling mediated prostatic response by paracrine WNT activity [28]. In rat neuronal differentiation from hippocampal neural stem and progenitor cells, BMP2 and BMP4 induced neurogenesis in a WNT-dependent manner. Furthermore, BMP also cooperated with WNT signaling to specify neuronal fate specification [29]. In addition, BMP2 could induce the WNT and planar cell polarity pathways to contribute to vascular smooth muscle motility [30]. A combinational treatment with BMP inhibitors (Noggin and Gremlin) and WNT inhibitor Frzb could give rise to the cranial muscle anlagen and promote myogenesis [31]. In a recent report, activation of BMP signaling was found to be required and together with WNT signaling to direct differentiated gut tube into colonic organoids [32]. In addition, Wei and colleagues recently reported that TGFbeta signaling desensitized basal cells from growth inhibitory effect of stromal WNT signaling in prostate epithelial progenitor [33]. Our results together suggested that BMP inhibitor promoted pancreatic differentiation possibly through two pathways: the well-known direct inhibitory effect for hepatic lineage and the indirect inhibitory effect for intestinal lineage mainly through inhibiting WNT signaling [21]. Our data indicated that BMP inhibition could decrease WNT activity and together promoted pancreatic differentiation, though how BMP inhibition resulted in decreased WNT activity during endodermal patterning remained to be dissected in future study.

Canonical WNT plays a key role in endoderm differentiation along with Nodal signal, whereas its upregulation is also required for gut lineage after endoderm [21]. Very recently, McCracken and colleagues reported that activated WNT signaling also promoted gastric fund differentiation in both rodents and human [34]. They observed that in E10.5 *Shh-cre*; *beta-catenin^fl/fl^* mouse embryos, beta-catenin was deleted in foregut-derived stomach budding region; the prestomach region was completely mispatterned with ectopic PDX1 expression [34]. This observation strongly suggested endogenous WNT signaling blocked pancreatic lineage specification. In addition, although WNT and BMP were generally considered to promote hindgut differentiation [21, 35], both of them played more complicated roles in the precise regulation of cell fate determination for different organ buds. We had determined the effect of WNT inhibitor only, compared to BMP inhibitor only and dual inhibitors (Figure 3). WNT inhibition alone indeed showed improved effect in terms of promoting pancreatic lineage marker expression, which was consistent with a recent report [36]; however, dual inhibition exhibited much better expression levels of pancreatic markers and more PDX1- and INSULIN-positive cells ([Supplementary-material supplementary-material-1]).

Taken together, these data combined with our study point out that precise combination, rather than one single signal self, determined the organ patterning. Understanding the precise signal crosstalk between WNT and BMP has greatly helped the directed pancreatic differentiation from human pluripotent stem cells.

## 5. Conclusion

We had identified that a combination of dual inhibition of the BMP and WNT signaling pathways could greatly promote pancreatic differentiation and beta cell generation from human pluripotent stem cells (i.e., human ESCs and iPSCs).

## Data Availability

The data used to support the finding of this study are included within the article.

## Supplementary Materials

Supplementary Materials The supplementary material files includes four supplemental figures (Figure S1-S4, including figure legends) and two supplemental tables (Table S1-S2). Figure S1: the PDX1/AFP/CDX2 expression under various BMP inhibitor treatment. Figure S2: the endodermal lineage differentiation upon treated with BMP inhibitor K02288. Figure S3: BMP inhibitor K02288 regulated pancreatic differentiation via decreased WNT activity. Figure S4: the beta cell generation upon treated with WNT inhibitor. Table S1: information of chemical compounds used in this study. Table S2: information of antibodies used in this study.
