# Supplementary material for: Dual Inhibition of BMP and WNT Signals Promotes Pancreatic Differentiation from Human Pluripotent Stem Cells
Source: Stem Cells Int. 2019 Dec 1;2019:5026793. doi: 10.1155/2019/5026793 (PMC6914911; doi:10.1155/2019/5026793)

Dual inhibition of BMP and WNT signals promotes pancreatic differentiation from human pluripotent stem cells

Mengtian Tan[1,2], Lai Jiang[1], Yinglei Li[1],Wei Jiang[1,3,#]

[1]Department of Biological Repositories, Zhongnan Hospital & Medical Research Institute, Wuhan University, Wuhan, China, 430071; [2]Affiliated Hospital of Hebei University of Engineering, Handan, China, 056038; [3]Hubei Provincial Key Laboratory of Developmentally Originated Disease, Wuhan, China, 430071

#To whom correspondence should be addressed. E-mail: jiangw.mri@whu.edu.cn

The supplementary materials include four supplemental figures (Figure S1-S4) and two supplemental tables (Table S1-S2).

Figure S1. The PDX1/AFP/CDX2 expression under various BMP inhibitors treatment.

A. Immunofluorescence results showing co-staining of pancreatic markers PDX1 and pancreatic NKX6-1, hepatic AFP, and intestinal CDX2 while treated with different BMP inhibitors (i.e., LDN193189 targeting ALK2/3, K02288 blocking ALK1/2/3/6, LDN214117 exclusively targeting ALK2, Dorsomorphin recognized as BMP inhibitor and AMPK inhibitor, and NOGGIN) during endodermal patterning. B. The percentage of PDX1-positive cells determined by intracellular flow cytometry in similar conditions to A (n=3).

Figure S2. The endodermal lineage differentiation upon treated with BMP inhibitor K02288.

A. Immunofluorescence results exhibited control group (No BMP inhibitor) and BMPi (shown for K02288) in PP stage with pancreatic markers PDX1, liver marker AFP, and intestinal marker CDX2. Scale bars for Enlargement inset, 50 μm. DAPI (blue) represented all cells for internal control. B. Flow cytometric analysis showed PDX1 expression in Control, BMPi (K02288). C. mRNA expression of *PDX1*, *NKX6-1*, *AFP*, and *CDX2* compared in control group and BMPi group (shown for K02288). ** represented for p-value < 0.01 and *** for p-value < 0.001 by student's *t* test (n=3). D. Co-staining PDX1 and NKX6-1 represented bona-fide pancreatic progenitors in BMPi (shown for K02288).

Figure S3. BMP inhibitor K02288 regulated pancreatic differentiation via decreased WNT activity.

A. The expression levels of WNT downstream genes (including *WNT3*, *TWIST1*, *AXIN2*, *MYC*) were analyzed between control group and BMPi group (using BMP inhibitor K02288) in PP stage (n=3). B-C. The protein of total beta-catenin and active beta-catenin were co-stained with PDX1 to show the intracellular localization (BMP inhibitor K02288).

Figure S4. The beta cells generation upon treated with WNT inhibitor.

Generated of INSULIN (red color) and PDX1 (green color) double-positive cells in Control group, BMPi group, WNTi group (XAV939) and “Dual i”. Scale bars for Enlargement inset, 50 μm.

Table S1. Information of chemical compounds used in this study.

**Compounds Name**	**Company**	**Catalog No.**	**Concentration**
Activin A	Peprotech	Cat #120-14	100 ng/ml
Wnt3a recombinant protein	R&D	Cat #5036-WN	50 ng/ml
ascorbic acid	Sigma	Cat #A4544	0.25 mM
KGF	R&D	Cat #251-KG	50 ng/ml
SANT1	Selleck	Cat #S7092	0.5 μM
TTNPB	Selleck	Cat #S4627	100 nM
PDBU	Sigma	Cat #4153/1	500 nM
K02288	Selleck	Cat #S7359	2 μM
LDN193189	Selleck	Cat #S2618	200 nM
LDN214117	Selleck	Cat #S7627	2 μM
Dorsomorphin	Selleck	Cat #S7840	2 μM
Noggin	Peprotech	Cat #120-10C	100 ng/ml
XAV-939	Selleck	Cat #S1180	2 μM
IWR-1	Selleck	Cat #S7086	2 μM
T3 (L-3,3,5-Triiodothyronine, Sodium Salt)	Sigma	Cat #T6397	1 μM
Repsox	Selleck	Cat #S7223	10 μM
YO-01027	Selleck	Cat #S2711	10 μM
zinc sulfate	Sigma	Cat #Z0251	10 μM
Trolox	EMD	Cat #648471	10 μM
heparin sodium	Selleck	Cat #S1346	10 μg/ml
R428	Selleck	Cat #S2841	2 μM
N-cys	Sigma	Cat #A9165	10 mM

Table S2. Information of antibodies used in this study.

**Antibodies Name**	**Company**	**Catalog No.**	**Concentration**
antibodies against beta-catenin	Cell Signaling Technology	Cat #8480	1:100

active beta-catenin	Cell Signaling Technology	Cat #8814	1:1000
goat anti-human PDX1	R&D	Cat #AF2419	1:200
GAPDH	Proteintech	Cat #60004-1-Ig	1:1000
rabbit anti-human PDX1	Abcam	Cat #ab47267	1:2000
rabbit anti-human AFP	Zhongshan	Cat #ZA0612	1:200
rabbit anti-human CDX2	Zhongshan	Cat #ZA0520	1:200
mouse anti-human CHGA	Zhongshan	Cat #ZM0076	1:200
rabbit anti-human CHGA	Zhongshan	Cat #ZA0507	1:200
rabbit anti-human GLUCAGON	Santa Cruz	Cat #sc-7779-R	1:50
mouse anti-human INSULIN	Santa Cruz	Cat #sc-8033	1:50
rabbit anti-human INSULIN	Santa Cruz	Cat #sc-9168	1:50
rat anti-C-Peptide	DSHB	Cat #GN-ID4	1:200
mouse anti-NKX6-1	DSHB	Cat # F55A12	1:100
mouse anti-pY489-beta-catenine	DSHB	Cat #PY489	1:100
rabbit anti-beta-catenin	Millipore	Cat #07-1653	1:200
mouse anti-Pro-INSULIN	DSHB	Cat #GS-9A8	1:50
goat anti-human SOX2	Santa Cruz	Cat #17320	1:50
TRITC conjugated donkey anti-rabbit IgG secondary antibodies	Jackson Immunoresearch	Cat #711-025-152	1:200
TRITC conjugated donkey anti-goat IgG secondary antibodies	Jackson Immunoresearch	Cat #705-025-147	1:200
TRITC conjugated donkey anti-mouse IgG secondary antibodies	Jackson Immunoresearch	Cat # 715-025-150	1:200
488-conjugated donkey anti-rabbit IgG secondary antibodies	Jackson Immunoresearch	Cat #711-095-152	1:200
488-conjugated donkey anti-goat IgG secondary antibodies	Jackson Immunoresearch	Cat #705-545-147	1:200
488-conjugated donkey anti-mouse IgG secondary antibodies	Jackson Immunoresearch	Cat #715-545-150	1:200
Cy™5 AffiniPure Donkey Anti-Goat IgG (H+L)	Jackson Immunoresearch	Cat #705-175-147	1:500

Figure S1.

A.

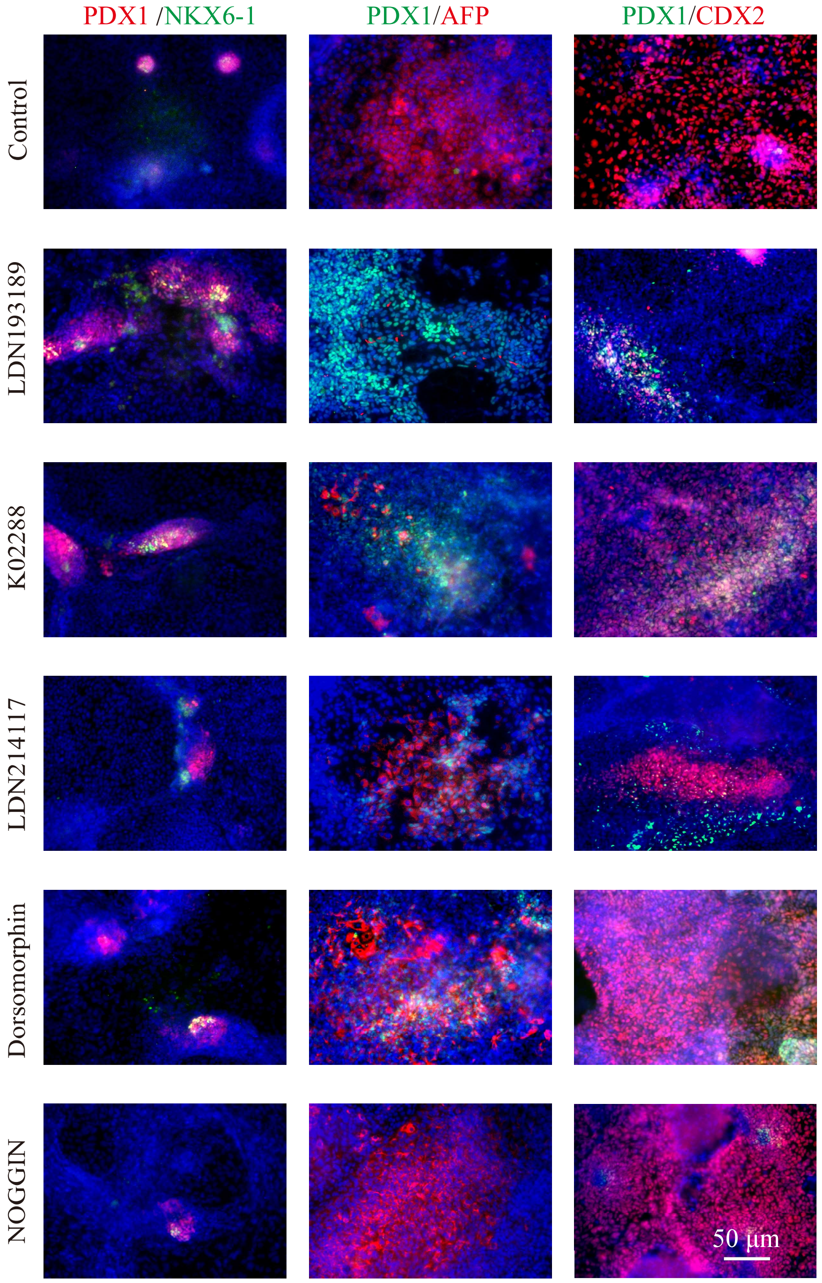
PDX1 /NKX6-1
PDX1/AFP
PDX1/CDX2
Control
LDN193189
K02288
LDN214117
Dorsomorphin
NOGGIN
50 µm

B.

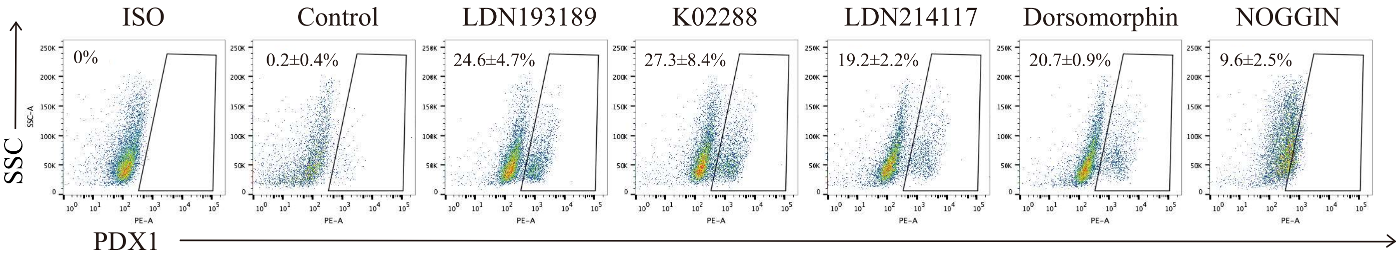
ISO
Control
LDN193189
K02288
LDN214117
Dorsomorphin
NOGGIN
0%
0.2±0.4%
24.6±4.7%
27.3±8.4%
19.2±2.2%
20.7±0.9%
9.6±2.5%
SSC
PDX1

Figure S2.

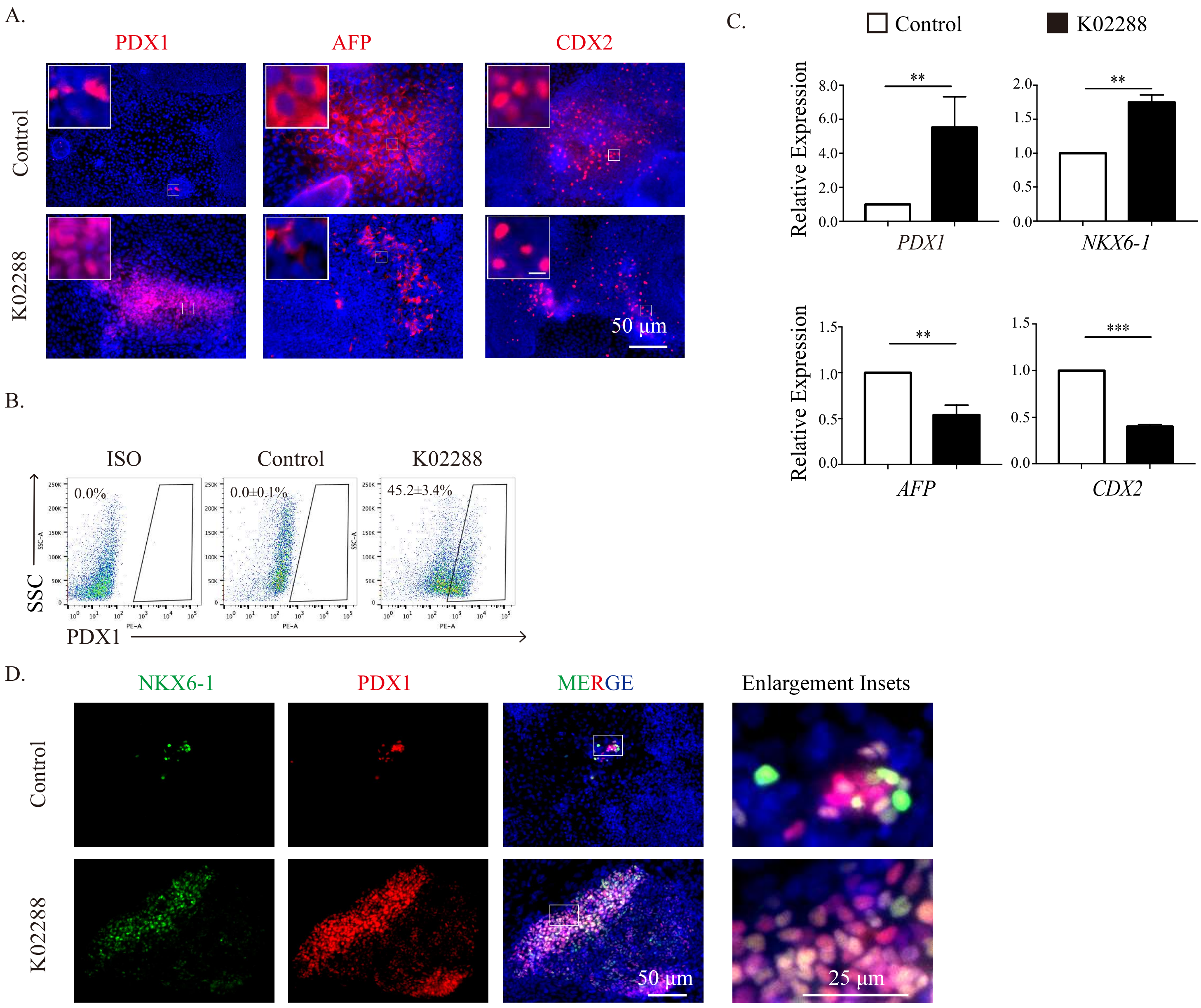
A.
PDX1
AFP
CDX2
Control
K02288
50 μm
B.
ISO
Control
K02288
0.0%
0.0±0.1%
45.2±3.4%
SSC
PDX1
C.
Control
K02288
Relative Expression
**
**
**
***
PDX1
NKX6-1
AFP
CDX2
D.
NKX6-1
PDX1
MERGE
Enlargement Insets
Control
K02288
50 μm
25 μm

Figure S3.

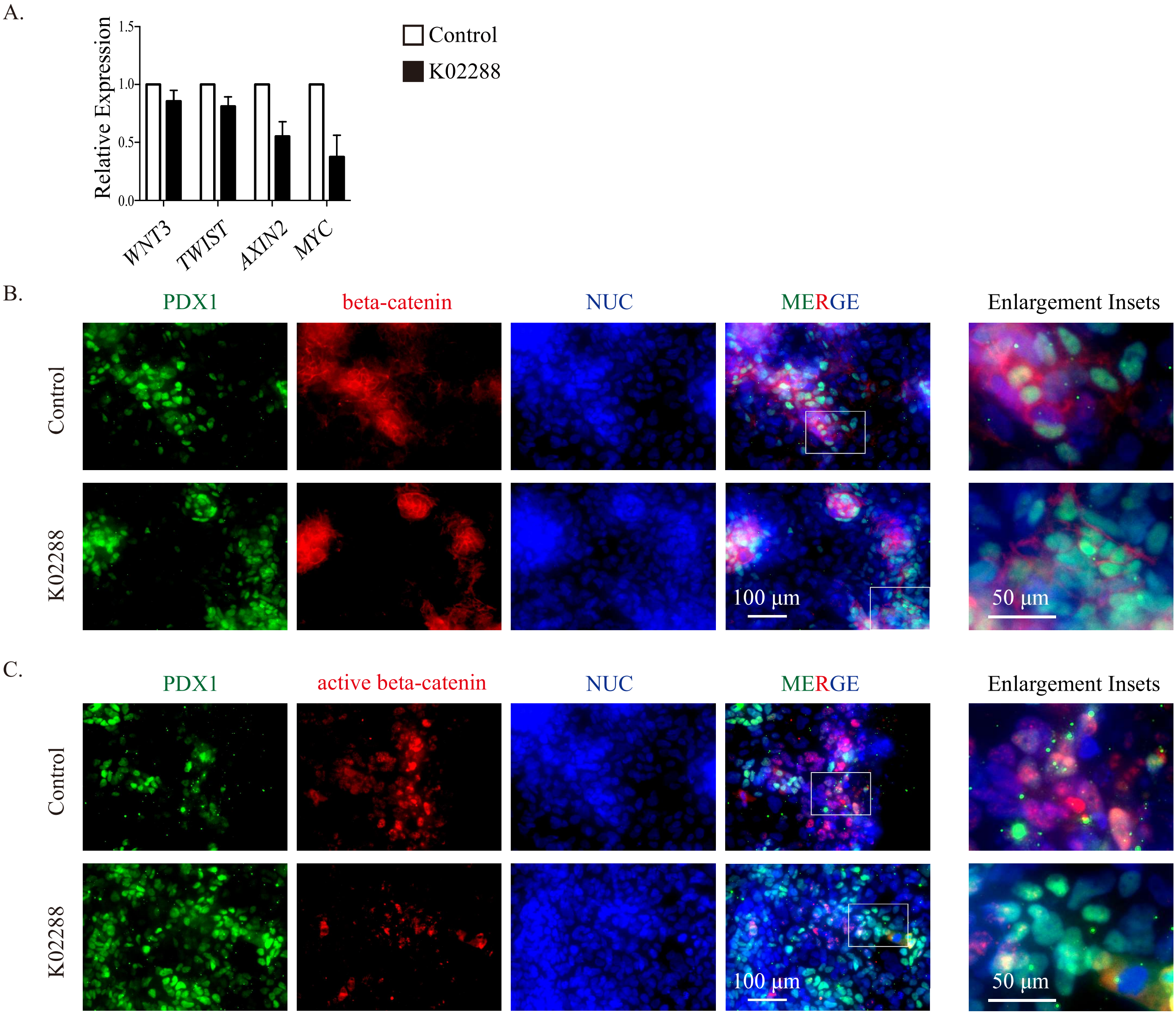
A.
Relative Expression
1.5
1.0
0.5
0.0
WNT3
TWIST
AXIN2
MYC
Control
K02288
B.
PDX1
beta-catenin
NUC
MERGE
Enlargement Insets
Control
K02288
100 µm
50 µm
C.
PDX1
active beta-catenin
NUC
MERGE
Enlargement Insets
Control
K02288
100 µm
50 µm

Figure S4.

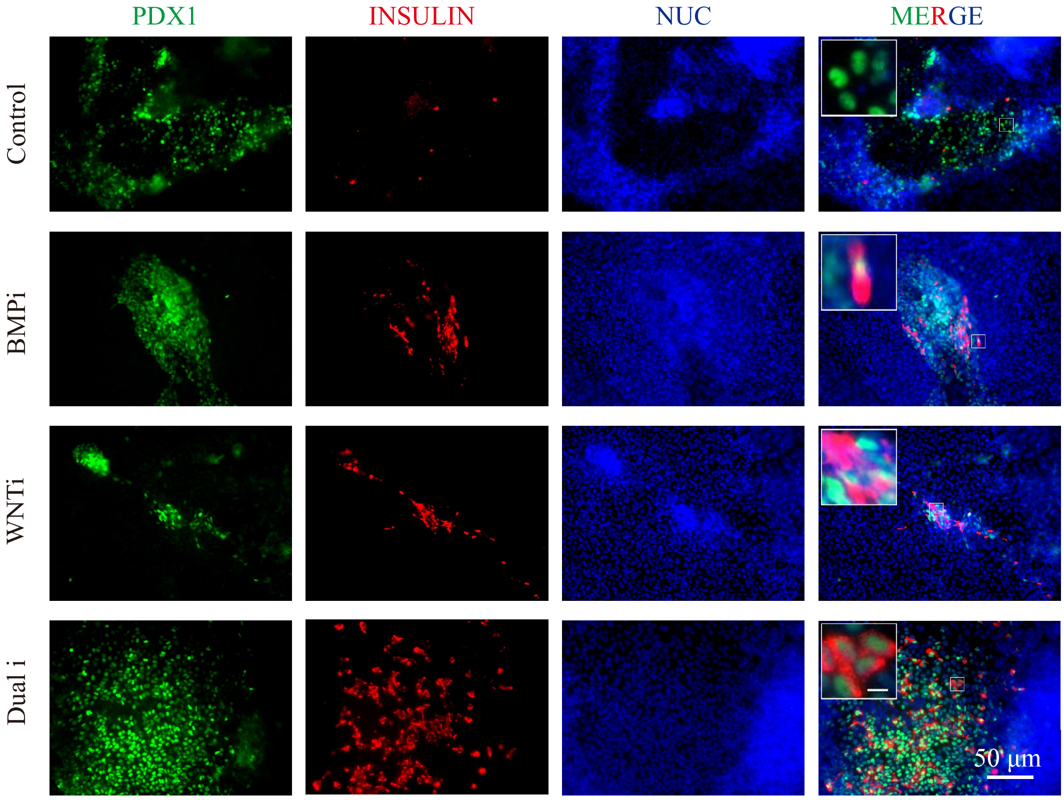

Supplement: Supplementary Materials — The supplementary material files includes four supplemental figures (Figure S1-S4, including figure legends) and two supplemental tables (Table S1-S2). Figure S1: the PDX1/AFP/CDX2 expression under various BMP inhibitor treatment. Figure S2: the endodermal lineage differentiation upon treated with BMP inhibitor K02288. Figure S3: BMP inhibitor K02288 regulated pancreatic differentiation via decreased WNT activity. Figure S4: the beta cell generation upon treated with WNT inhibitor. Table S1: information of chemical compounds used in this study. Table S2: information of antibodies used in this study. [file 5026793.f1.pdf]
